# Retraining Convolutional Neural Networks for Specialized Cardiovascular Imaging Tasks: Lessons from Tetralogy of Fallot

**DOI:** 10.1007/s00246-020-02518-5

**Published:** 2021-01-04

**Authors:** Animesh Tandon, Navina Mohan, Cory Jensen, Barbara E. U. Burkhardt, Vasu Gooty, Daniel A. Castellanos, Paige L. McKenzie, Riad Abou Zahr, Abhijit Bhattaru, Mubeena Abdulkarim, Alborz Amir-Khalili, Alireza Sojoudi, Stephen M. Rodriguez, Jeanne Dillenbeck, Gerald F. Greil, Tarique Hussain

**Affiliations:** 1 Department of Pediatrics, UT Southwestern Medical Center, 1935 Medical District Dr, Dallas, TX 75235 USA; 2 Department of Radiology, UT Southwestern Medical Center, 1935 Medical District Dr, Dallas, TX 75235 USA; 3 Division of Cardiology, Children’s Health Children’s Medical Center Dallas, Dallas, TX USA; 4 Circle Cardiovascular Imaging, Calgary, AB Canada; 5 Pediatric Cardiology, Department of Surgery, Pediatric Heart Center, University Children’s- Hospital Zurich, Zurich, Switzerland; 6 King Faisal Specialist Hospital and Research Centre, Jeddah, Saudi Arabia; 7 Department of Pediatrics, LeBonheur Children’s Hospital and University of Tennessee, Memphis, TN USA

**Keywords:** Convolutional neural network, Congenital heart disease, Tetralogy of fallot, Ventricular contouring, Machine learning

## Abstract

Ventricular contouring of cardiac magnetic resonance imaging is the gold standard for volumetric analysis for repaired tetralogy of Fallot (rTOF), but can be time-consuming and subject to variability. A convolutional neural network (CNN) ventricular contouring algorithm was developed to generate contours for mostly structural normal hearts. We aimed to improve this algorithm for use in rTOF and propose a more comprehensive method of evaluating algorithm performance. We evaluated the performance of a ventricular contouring CNN, that was trained on mostly structurally normal hearts, on rTOF patients. We then created an updated CNN by adding rTOF training cases and evaluated the new algorithm’s performance generating contours for both the left and right ventricles (LV and RV) on new testing data. Algorithm performance was evaluated with spatial metrics (Dice Similarity Coefficient (DSC), Hausdorff distance, and average Hausdorff distance) and volumetric comparisons (e.g., differences in RV volumes). The original Mostly Structurally Normal (MSN) algorithm was better at contouring the LV than the RV in patients with rTOF. After retraining the algorithm, the new MSN + rTOF algorithm showed improvements for LV epicardial and RV endocardial contours on testing data to which it was naïve (N = 30; e.g., DSC 0.883 vs. 0.905 for LV epicardium at end diastole, *p* < 0.0001) and improvements in RV end-diastolic volumetrics (median %error 8.1 vs 11.4, *p* = 0.0022). Even with a small number of cases, CNN-based contouring for rTOF can be improved. This work should be extended to other forms of congenital heart disease with more extreme structural abnormalities. Aspects of this work have already been implemented in clinical practice, representing rapid clinical translation. The combined use of both spatial and volumetric comparisons yielded insights into algorithm errors.

**Supplementary Information:**

The online version of this article (10.1007/s00246-020-02518-5) contains supplementary material, which is available to authorized users.

## Introduction

Early uses of neural networks in medicine used hundreds of thousands of cases to show physician-level accuracy in image-based diagnoses, e.g., for skin cancer [1] and diabetic retinopathy [2]. Since then, in cardiac imaging, there have been numerous uses of convolutional neural networks (CNNs) and other machine learning algorithms to both perform specific tasks and to generate new knowledge [3–7]. Most of these examples have hundreds of cases and primarily focus on adult left ventricles, though there are examples that focus on the right ventricle [8, 9].

Cardiovascular magnetic resonance (CMR) imaging is a non-invasive and safe imaging modality, and its use is growing in congenital heart disease. CMR is generally considered to be the gold standard imaging modality for ventricular volume and function measurement, as calculating these parameters can be done with minimal spatial assumptions as compared to echocardiography [10–12]. Tetralogy of Fallot is the most common indication for CMR in congenital heart disease [13–15]; one reason for this is that the right ventricle (RV) specifically has a complex 3-dimensional shape that is difficult to interrogate well with 2D imaging methods, and RV volumes and function are key indicators for performing pulmonary valve replacement in patients with repaired tetralogy of Fallot (rTOF) [16] and may relate to outcomes [17].

Ventricular volumes and function are calculated through contouring, where the endocardial and epicardial surfaces of the ventricular muscle are outlined, then summed using the method of disks [18]. Contouring the ventricles to determine volumes and function is thus an integral part of CMR post-processing, but contouring is a time-consuming process and has inherent intraobserver and interobserver variability. For an adult CMR with a normal-shaped heart, contouring has been reported to take about 20 min [19]; for a patient with complex congenital heart disease, manual contouring undoubtedly takes longer. Thus, this task is an ideal target for automation.

Recently, machine learning techniques, namely neural networks, have been developed to automate ventricular contouring. For instance, Bai et al. [19] recently used 4875 CMR studies from the UK Biobank (UKBB) to train a CNN to automatically generate ventricular contours. They used 3,975 subjects for training the neural network, 300 validation subjects for tuning model parameters, and finally, 600 test subjects for evaluating performance. Suinesiaputra et al. [20] used two versions of a different automated method to analyze UKBB CMRs. The UKBB CMR dataset consists of mainly healthy adults in the UK (mean 63.4 ± 7.56 years, 52% female, source: http://biobank.ndph.ox.ac.uk/showcase/field.cgi?id=21003), with generally structurally normal hearts. The protocol for the UKBB has been described [21], as has the post-processing [22].

As noted, most machine learning CMR contouring tools are trained on adults with structurally normal hearts. Other approaches to addressing the issue of training algorithms with small numbers of cases of congenital heart disease also have been proposed [23], though not many.

Given the importance of CMR values, especially RV values, on decision making for children and adults with rTOF, it is vital to improve contouring in congenital heart disease to reduce contouring time and potentially reduce variability. Thus, we evaluated a CNN that was trained on UKBB data combined with selected other pathologies such as hypertrophic cardiomyopathy, but no congenital heart disease. We evaluated its performance on left ventricular (LV) and RV contouring in rTOF, testing LV epicardium (LV epi), LV endocardium (LV endo), and RV endo, at end diastole (ED) and end systole (ES), as these are the contours most commonly drawn clinically. We hypothesized that the algorithm would be worse at contouring the RV than the LV given the more complex shape of the RV and higher likelihood of RV dilation in rTOF, and that adding rTOF training data for the algorithm would improve both LV and RV contouring in rTOF. Our study is novel because we examined a potential method to solve the problem of small case numbers in pediatric cardiology. By using an existing algorithm, trained on a large number of adult datasets, testing it on congenital CMRs, and then improving it with a small number of congenital CMRs, we can evaluate whether this strategy could be viable for other uses of machine learning in pediatric cardiology as well. Further, we evaluated the performance of the algorithms using both spatial- and volumetrics.

## Materials and Methods

### Patient Datasets

Patients were included that had a diagnosis of tetralogy of Fallot with pulmonary stenosis or atresia that had undergone initial repair and underwent a follow-up CMR study at our institution between 1/2016 and 7/2019. Patients with tetralogy of Fallot with absent pulmonary valve were excluded. This study was performed under UTSW IRB STU 122017–037.

The rTOF cases were divided into two groups by time, with the earlier cases assigned to the training dataset. This training dataset was used to evaluate the initial CNN for use in rTOF, and then used to retrain the CNN. The more recent cases were assigned to the testing dataset and were used to compare performance of the initial (mostly structurally normal, MSN) and revised (MSN + rTOF) contouring algorithms.

### Typical CMR Parameters

CMR was performed on a 1.5T Ingenia scanner (Philips Healthcare, Best, The Netherlands) using a 32-channel torso phased-array digital receiver coil. ECG gated balanced cine steady-state free precession images (bSSFP) were obtained in a short-axis stack of 9–13 slices from above the atrioventricular valves to the apex, in 30 phases per cardiac cycle with a slice thickness of 8–10 mm, no gap, field of view between 272 mm × 272 mm and 390 mm × 390 mm, echo time 1.11–1.68 ms, temporal resolution at a median of 34.5 ms (25.3–50 ms). The cines were performed with breath-holding technique if possible, otherwise, 2 signal averages were used in combination with respiratory bellows gating for patients who could not perform a breath hold. There were no changes to the bSSFP sequence through the study, and hence the same sequence was used for patients in both the training and testing datasets.

### Manual CMR Ventricular Contouring

Manual contouring of the rTOF training and testing datasets was done for clinical purposes using standard post-processing practices as described by Fratz et al. [18] using cvi42 version 5.9 (Circle Cardiovascular Imaging, Calgary, Alberta, Canada). As is standard, flow data were incorporated as internal check to ensure accuracy of ventricular contours. All clinical contouring was performed by readers with > 1 year of CMR experience and checked by readers with > 5 years CMR experience. For intraobserver and interobserver variability calculations in the testing dataset, half the cases were recontoured by the initial person who did the contours (VG), while interrater contours were performed by another expert with 6 years CMR experience (BEUB).

### Initial Convolutional Neural Network (CNN) Algorithm

The machine learning algorithm employed herein to predict ventricular contours was a CNN based on the U-net architecture [24]. The CNN was trained to associate pixel intensities of a CMR image to segmentation maps corresponding to the desired ventricular contours. During the training stage, the model parameters of the CNN were optimized to reduce an energy function computed using the pixel-wise cross-entropy loss function, which penalizes the CNN when it does not correctly predict the segmentation label of a given pixel.

The initial CNN was trained on the UKBB CMR dataset on ~ 5000 CMR studies, as well as a set of 100 pathologic CMR studies including cases of hypertrophic cardiomyopathy, dilated cardiomyopathy, and myocardial infarction, but no rTOF cases. Given that these are mostly normal hearts, we labeled this algorithm the Mostly Structurally Normal (MSN) algorithm. The MSN algorithm is available in Circle cvi42 version 5.9 (Circle Cardiovascular Imaging, Calgary, Canada).

The CNN was trained on images with spatial resolution of 198 × 198 pixels with a pixel spacing of 1.855 × 1.855 mm/pixel. This allowed the network to be trained on images with varying field-of-views and acquisition specific resolution. Batch normalization layers are used to standardize the intensity of input images. To increase the generalizability of the network, image augmentation techniques such as rotation, scaling, translation, and mirroring were applied to the input data. Early stopping was also used to avoid overfitting. No other regularization techniques, i.e., dropout or weight decay, were used during the training of this network.

The cvi42 software uses a proprietary, heuristic-based algorithm to post-process the results of the CNN into contours that reside in a 4 × 4 subpixel space of the original input image. All results reported in this paper are reported on the post-processed cvi42 contours.

### Training a New Convolutional Neural Network (CNN) Algorithm

The manual rTOF contours from the training dataset were then used to retrain the MSN algorithm to yield the MSN + rTOF algorithm. This was accomplished by incorporating the rTOF training data into the pool of MSN training data. During training stage of the MSN + rTOF algorithm, the number of rTOF cases was oversampled in each training epoch to ensure that the CNN does not learn to ignore the rTOF cases in the early stages of training since the > 5000 MSN data vastly outnumber the rTOF instances. The rTOF images were processed to match the spatial resolution for which the MSN network was trained. Aside from changes to the training data, the exact same CNN architecture and optimization parameters were used in the MSN and MSN + rTOF experiments. The same cvi42 post-processing algorithm was used for this network.

### Evaluation of Contouring Performance—Spatial Metrics

The manually generated contours used for clinical reporting were considered the gold standard contours and thus were the basis for all comparisons.

Contours were analyzed using both spatial- and volumetrics. In terms of spatial metrics, the Dice Similarity Coefficient (DSC) represents spatial overlap in three dimensions and is calculated using the formula DSC = (2*(A ∩ B)/(A + B)) where A ∩ B represents the volume of the spatial overlap, and A and B represent the volumes of the original clinical ventricular contour and comparison contour, respectively [25]. A DSC of 1 represents perfect spatial overlap, while 0 means no spatial overlap at all. The Hausdorff distance (HD) is a spatial distance measure and is the maximum distance of a point on one contour to the nearest point on the other contour [26]; we took the mean of these values across all slices. Given that the Hausdorff distance is sensitive to outliers, we also used the Average Hausdorff Distance (AVD), which is the Hausdorff distance averaged over all points on both contours. We used the mean AVD over all slices [26].

### Evaluation of Contouring Performance—Volumetrics

The ventricular end-diastolic volumes (EDV), end systolic volumes (ESV), and ejection fractions (EF) were compared between contouring methods by assessing the absolute value of the percentage difference in volume or EF as compared to the manually calculated volumes (%error).

### Statistical Comparisons

For patient characteristics, Mann–Whitney and t-tests with Welch’s correction were used. For spatial comparisons, Wilcoxon signed-rank tests were used. For volumetric comparisons, Wilcoxon matched-pair signed-rank test, linear correlation, and Bland–Altman analyses were performed. In all cases, *p* < 0.05 was considered significant. Statistical analyses were performed using GraphPad Prism version 8.1 (GraphPad Software, San Diego, CA).

### Determination of Intra- and Interrater Variability of Contours

To determine intra- and interrater variation of contours, half of the patients in the testing dataset had contours redrawn by the original contourer (VG), as well as another expert in CMR with 6 years’ experience (BEUB). Intra- and interrater spatial- and volumetrics were calculated.

### Examination of Sources of Error

Patients were sorted by worst performance on spatial and volumetric measures on both MSN and MSN + rTOF, and those whose performance declined the most from MSN to MSN + rTOF. The six cases that appeared most commonly in these lists were manually reviewed to find patterns that could explain poor algorithm performance.

In addition, we analyzed the data again after removing algorithm-generated contours in slices where there were no manual contours.

## Results

### Patient Characteristics

The rTOF training dataset initially consisted of 59 cases, but the CNN was designed to train on cases where both LV and RV contours were in the same cardiac phase, so 57 cases could be used for diastole, and 31 for systole. The rTOF testing dataset was initially 32 cases, but for similar reasons only 30 were used. The technical exclusion rate is similar to other such studies [20]. Patient characteristics are shown in Table 1. There were no significant differences in age, body surface area (BSA), heart rate, at time of CMR, or number of studies with breath-holding versus signal averages, between the training and testing datasets.

**Table 1:** Patient characteristics

	N	Females (%)	Age at CMR (yr) (Median, IQR)	BSA at CMR (m^2^) (mean ± stdev)	Heart rate at CMR (bpm) (mean ± stdev)	Breath-held imaging
Training	57	31 (54%)	13.5 (10.0,17.5)	1.42 ± 0.45	77.2 ± 12.1	44 (77%)
Testing	30	19 (63%)	13.9 (11.7,18.0)	1.44 ± 0.52	74.4 ± 16.2	23 (77%)
*p*-value			0.766 (Mann Whitney)	0.82 (t-test with Welch’s correction)	0.39 (t-test with Welch’s correction)	

This table shows the age, body surface area, and heart rate for the patients with repaired tetralogy of Fallot (rTOF) that were used in the study. There were no significant differences between the training and testing datasets in age, BSA, heart rate, or number of cases done with breath-holding

### Performance of MSN Algorithm on Training rTOF CMR Data

**Fig. 1:**
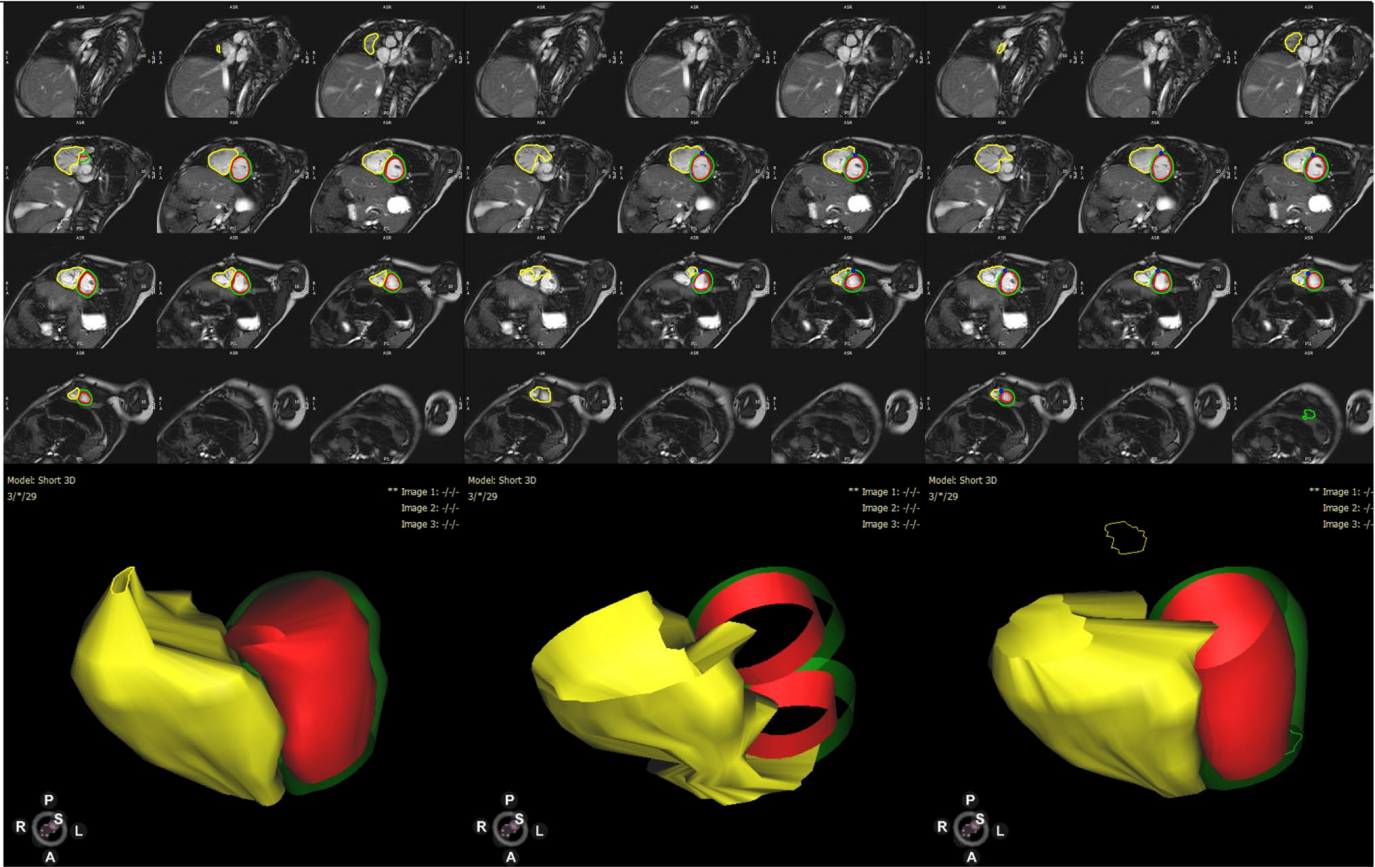
Cardiac magnetic resonance contours for a patient with repaired tetralogy of Fallot and right ventricular dilation, who showed significant improvement in RV contours after training. End diastole is shown, with manual contours on the left, contours derived from the initial MSN algorithm in the middle, and retrained MSN + rTOF algorithm on the right. Note slice selection errors with both MSN and MSN + rTOF methods. 3D representations of the ventricular contours are shown below. Left ventricular endocardial contours are in red, left ventricular epicardial in green, and right ventricular endocardial in yellow

**Fig. 2:**
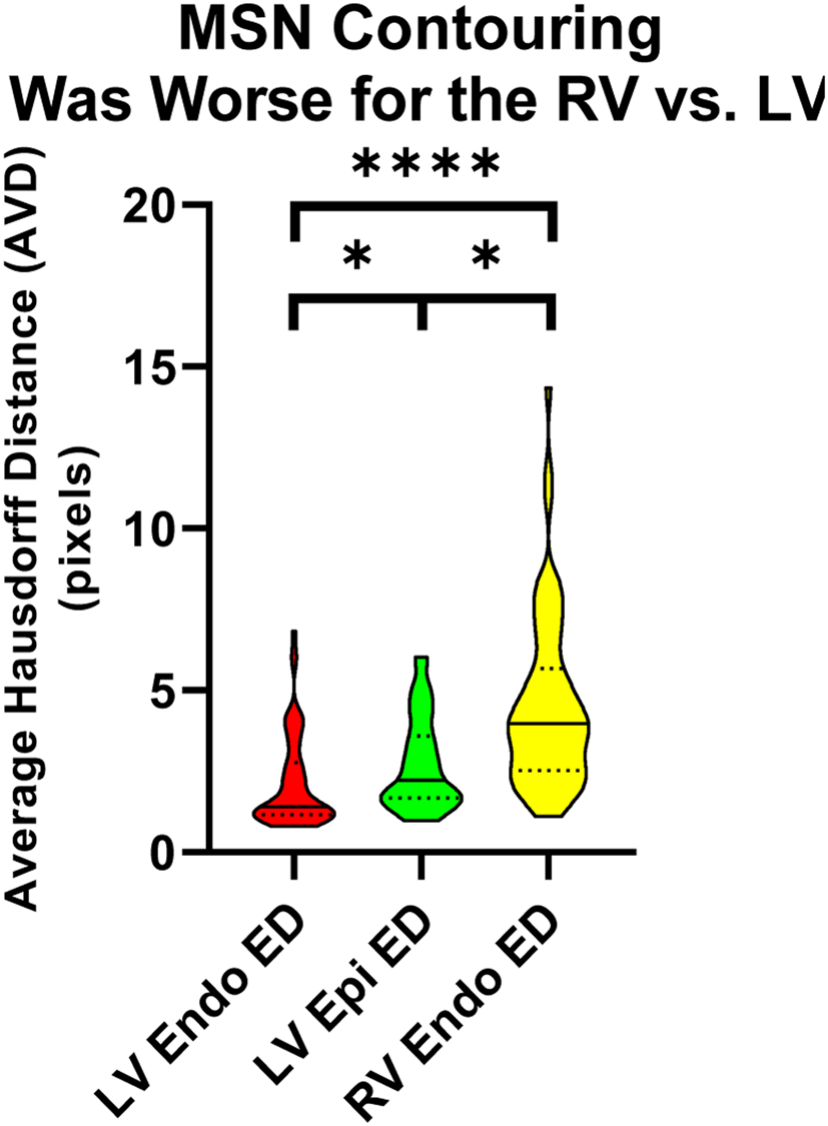
Spatial MSN performance on the training dataset. As hypothesized, the RV endocardial contours generated by the MSN algorithm were consistently worse than the LV contours. Representative ED AVD data are shown. Violin plots with LV endo contours in red; LV epi contours in green; and RV endo in yellow. Bars represent median and IQR. * represents *p* < 0.05; **** represents *p* ≤ 0.0001

**Fig. 3:**
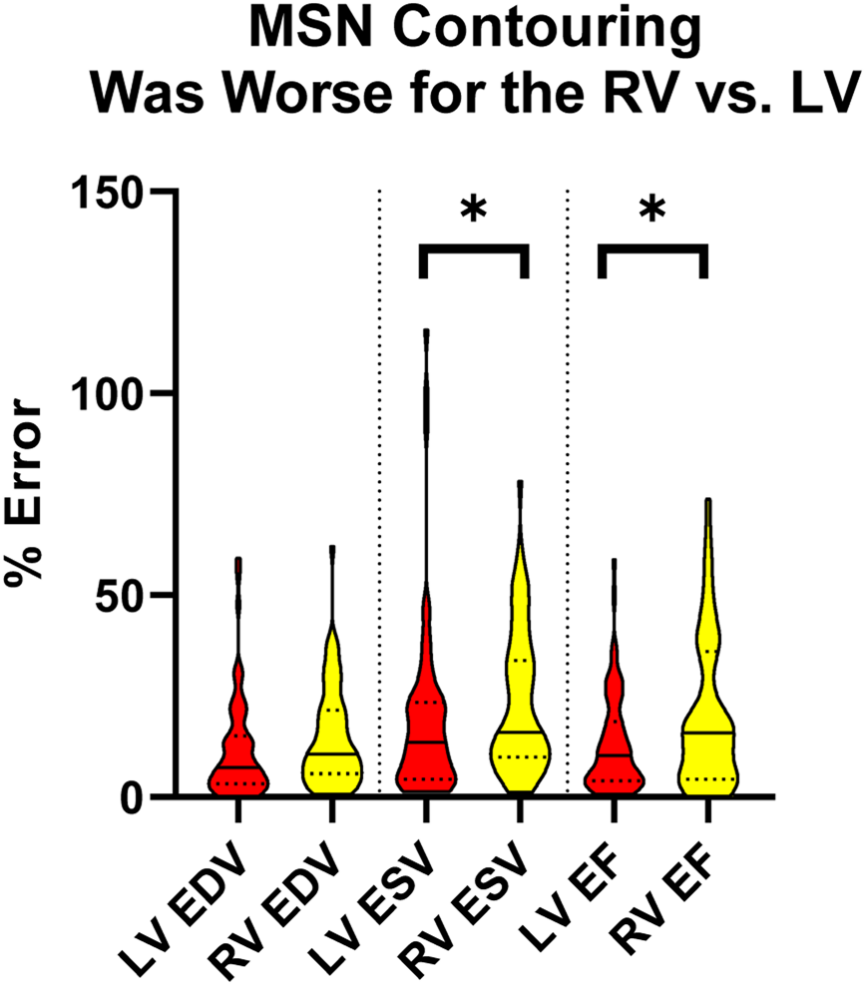
Volumetric MSN performance on the training dataset is worse for the RV. EDV is shown on the left, ESV in the middle, and EF on the right. LV volumes with red and RV volumes in yellow. Solid line represents median and dotted lines IQR. * represents *p* < 0.05

We initially tested how well the MSN algorithm contoured the LV endo, LV epi, and RV endo for patients with repaired rTOF (Fig. 1). The spatial results are summarized in Supplemental Table 1 and volumetric results in Supplemental Table 2. In short, the RV endo contours generated by the MSN algorithm were consistently worse than the LV contours when evaluated with DSC and HD; when using AVD, the LV endo were better than the LV epi as well. ED AVD results are shown in Fig. 2. The MSN algorithm was also worse at contouring the RV than the LV for ESV and EF, as assessed by %error compared to the manual contouring (Fig. 3).

### Testing the Retrained MSN + rTOF Algorithm on New Testing Data

Next, we evaluated the performance of the MSN + rTOF algorithm on new rTOF cases (testing dataset) and compared it to the standard MSN algorithm. Thirty cases were used and analyzed using both spatial- and volumetrics.

**Fig. 4:**
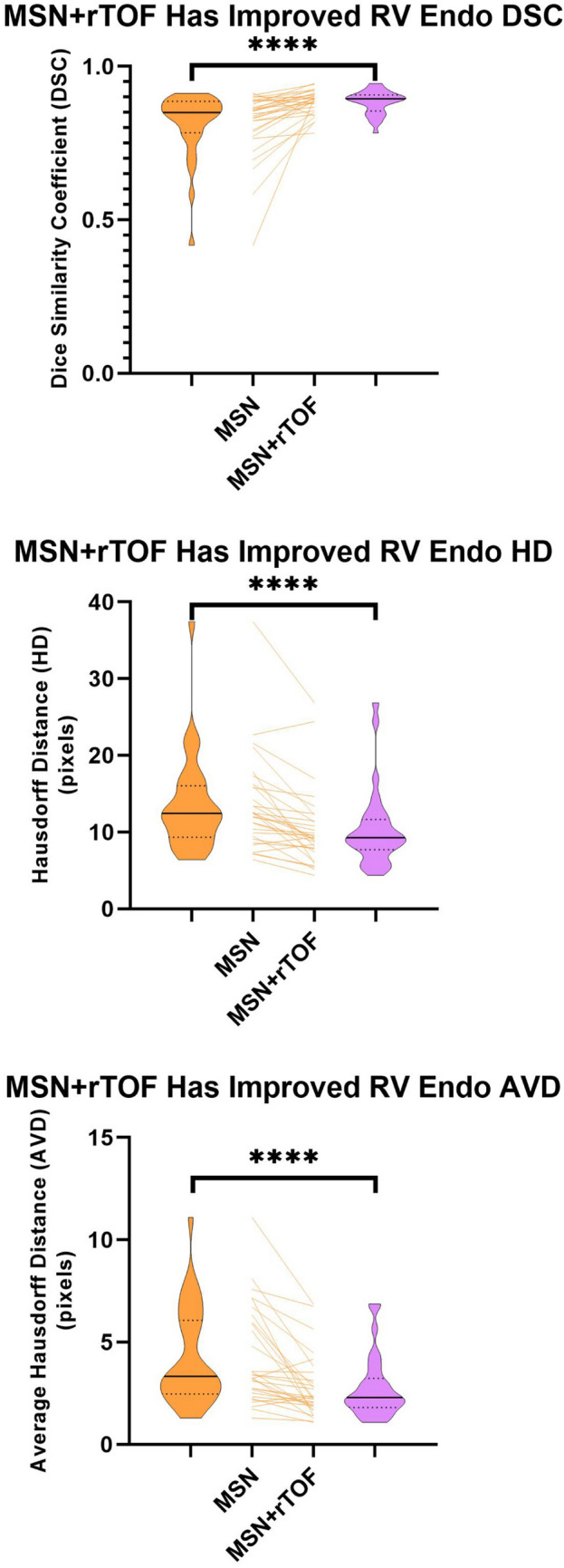
MSN vs. MSN + rTOF algorithm spatial performance on the testing dataset. Example data are shown for RV endo contours, with DSC (top), HD (center), and AVD (bottom). Violin plots are shown with MSN on the left (orange) and MSN + rTOF on the right (purple), and changes for individual cases are shown in the middle. Solid line represents median and dotted lines IQR. * represents *p* < 0.05; ** represents *p* < 0.01; *** represents *p* < 0.001; **** represents *p* ≤ 0.0001

Regarding spatial metrics, LV epi and RV endo contours improved from MSN to MSN + rTOF in all three evaluation metrics (DSC, HD, AVD), with LV endo also having an improved DSC (Fig. 4, Supplemental Table 3).

**Fig. 5:**
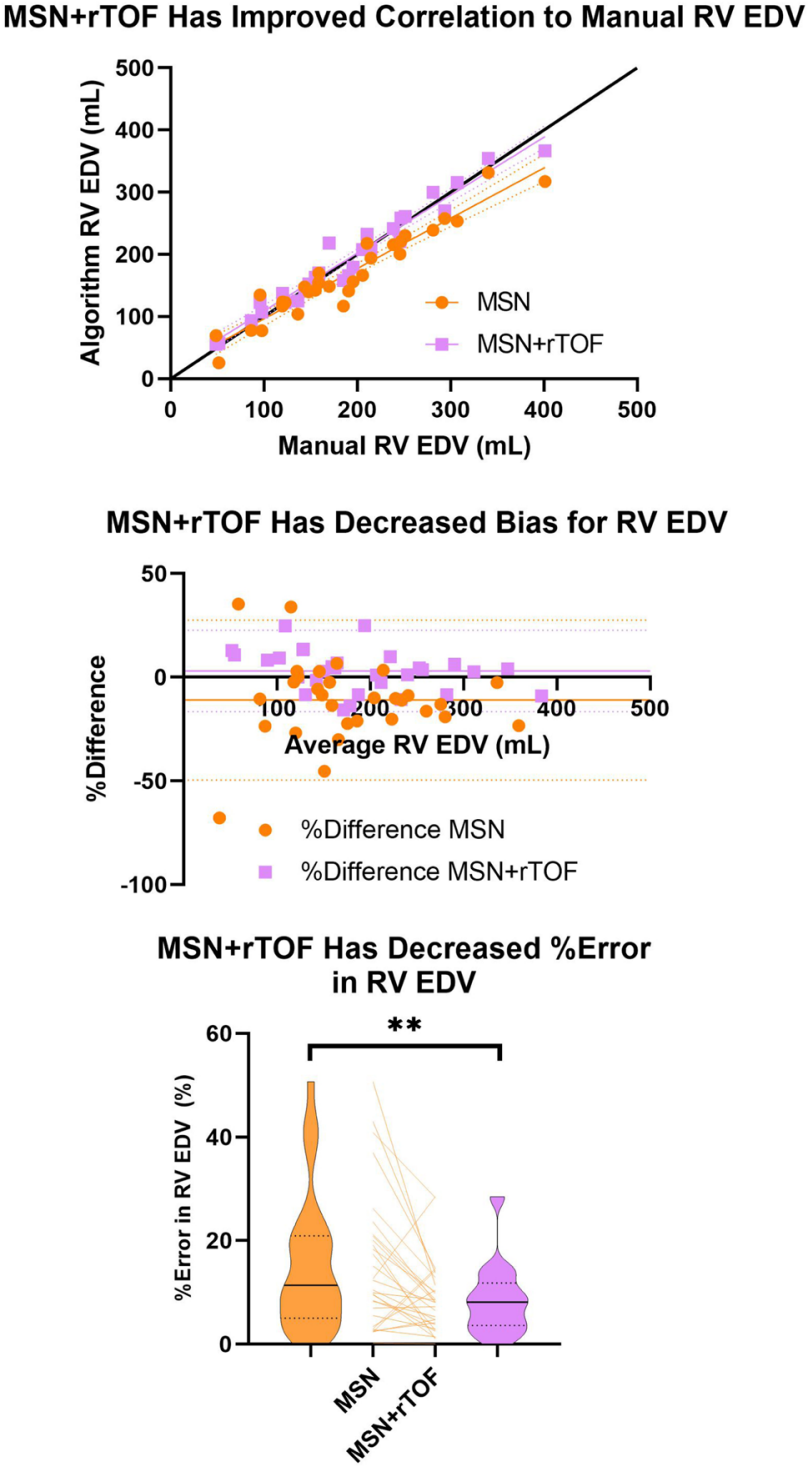
Volumetric comparisons of MSN and MSN + rTOF algorithms. The top panel shows correlation of algorithmic RV EDV and manual RV EDV. The line of identity, best-fit lines, and best-fit line errors are shown. MSN + rTOF showed significantly improved correlation with manual volumes compared to MSN (*p* = 0.0459). The middle panel shows the Bland–Altman analysis of MSN and MSN + rTOF RV EDV compared to the manual contours. The bottom panel shows a violin plot with MSN on the left and MSN + rTOF on the right, and changes for individual cases shown in the middle. Solid line represents median and dotted lines represent IQR. ** represents *p* < 0.01

Regarding volumetrics, MSN + rTOF showed an improved correlation with the current gold standard manual RV EDV. Also, LV ED mass and RV EDV contoured by the MSN + rTOF algorithm showed reduced %error compared to the MSN algorithm. In all other cases, there were no significant differences in correlation and %error. Example data for RV EDV are shown in Fig. 5. Full data are shown in Supplemental Table 4.

### Comparison of MSN + rTOF Algorithm to Intra- and Interrater Contours

Intrarater and interrater spatial and volumetric results are shown in Supplemental Tables 5 and 6, respectively. In brief, MSN + rTOF spatial performance was comparable to intra- and interrater contours for all except LV endo and epi as measured by HD and AVD. MSN + rTOF volumetric performance was significantly worse for the LV but generally within intra- and interrater contours for the RV.

### Examination of Sources of Error

Of the six cases that were identified as the worst-performing, the most common issue was the algorithm drawing contours on inappropriate slices, i.e., the algorithms would contour a part of the atria as part of a ventricle. One poorly performing patient did not have a clear pulmonary valve after transannular patch repair and the MSN + rTOF algorithm included more of the right ventricular outflow tract, and another had rTOF as well as hypertrophic cardiomyopathy.

When we removed the algorithm-generated contours in slices where there were no manual contours, the overall results were generally similar, though the magnitude of errors were lower (Supplemental Tables 7 and 8).

## Discussion

In this paper we evaluated a machine learning algorithm, a convolutional neural network (CNN), for ventricular contouring that was trained on mostly structurally normal hearts. We showed that this MSN algorithm did better for the LV than the RV in patients with rTOF across a number of different metrics, using both spatial and volumetric assessments by design. We then used those cases to create an improved algorithm MSN + rTOF. This improved algorithm showed clear benefits with spatial metrics, and improvements with some volumetric measures as well. This suggests that the MSN + rTOF algorithm learned improvements that generalize to studies beyond the training dataset.

Most automated analysis tools are developed for structurally normal hearts, and fewer are designed specifically for congenital heart disease, likely because training these algorithms initially requires a significant volume of cases. Our work shows that even with small numbers of cases, an already established algorithm can be expanded to use in other clinical scenarios. Also, the inclusion of rTOF cases to generate the MSN + rTOF algorithm did not degrade performance on structurally normal hearts (industry-level testing data not shown), thus expanding the clinical utility of the algorithm.

As the field of pediatric cardiology will always face the issue of lower numbers and clinical heterogeneity, these findings are of clinical importance. This work should encourage further investigation of modifying solutions to adult problems to fit the needs of patients with congenital heart disease and to find avenues to address more niche clinical needs that have smaller patient or image volumes.

### Comparison of MSN + rTOF Algorithm to Intra- and Interrater Contours

The performance of the MSN + rTOF algorithm was in general worse than intra- and interrater contours for the LV and in general not significantly worse than intra- and interrater contours for the RV. This is likely due to the higher intrarater differences in RV contouring. These findings are similar to Blalock et al. [27] who reported repeatability between two observers of end-diastolic ventricular volumes at 15% in rTOF. Mooij et al. [28] showed that in 20 rTOF CMRs, the RV EDV mean volume difference was 6.3% with a coefficient of variability, calculated as the standard deviation interrater difference divided by the mean RV EDV, of 4.8%. The MSN + rTOF RV EDV coefficient of variability was 9.6%, suggesting there is still optimization to be done.

### Comparison to Previous Studies

There have been multiple studies examining automated contouring in adult CMR studies (e.g., Bai et al. [19],Suinesiaputra et al. [20]), and even MICCAI challenges (e.g., Feng et al. [29],Yang et al. [30]). There are also deep learning approaches for combined segmentation and disease classification using CMR [31] and echocardiography [32]. However, there is limited knowledge about using neural network contouring methods for congenital CMR. Diller et al. [33] did use deep learning methods to segment and classify transthoracic echocardiograms from patients with transposition of the great arteries after atrial switch procedure or congenitally corrected transposition of the great arteries (both of which have a systemic right ventricle), and Diller et al. [34] used deep learning to de-noise transthoracic echocardiograms for congenital heart disease. Pace et al. [23] proposed iterative methods to overcome the challenge of small numbers of cases. These studies, along with the current study, show that there is clearly a role for deep learning and other automated approaches to improve congenital heart disease cardiac imaging, despite the fact that there will be fewer studies than adult heart disease. We believe that given the limitations of clinical heterogeneity and small overall numbers, advanced analytical techniques for image analysis in congenital heart disease might even be more important than in adult heart disease [35, 36].

### Use of Multiple Contour Performance Metrics

We purposely chose to use multiple spatial (DSC, HD, and AVD) and volumetric (e.g., %error of EDV, EF) measures to evaluate the performance of the algorithms on our datasets. DSC focuses solely on spatial overlap and thus is susceptible to overestimating performance if the central volume of the contours is correct despite the edges being less accurate. Because the shape of the ventricles is important for ventricular contouring as well as the volume, spatial distance-based metrics, namely, HD and AVD, were also used [26]. Ventricular volumetrics (including ejection fraction) were used because these are clinically relevant metrics, but subject to the limitation that contours with different shapes can still yield similar volumes. Combining both types of metrics revealed insights into errors generated by the algorithms, which may not have been obvious with only one type of metric. We suggest this more comprehensive methodology be used going forward when evaluating the performance of contouring algorithms.

### Improving Sources of Error

**Fig. 6:**
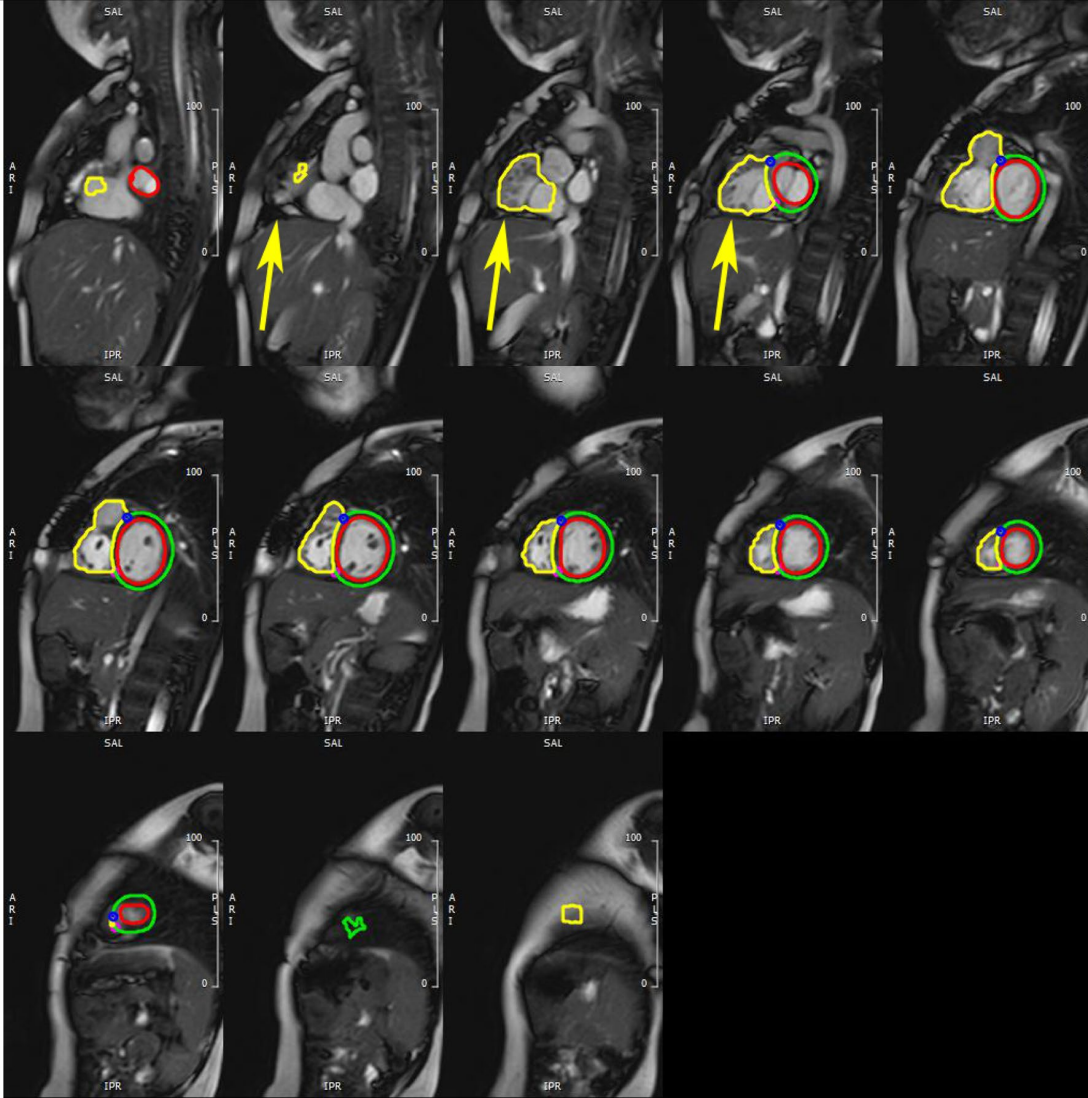
Shown here are the MSN + rTOF contours at ED. This patient has a significantly dilated RV, with the “shoulder” extending more basally past the tricuspid valve plane (yellow arrows), necessitating extension of the short-axis slices basally past the mitral and tricuspid valve plane. The MSN + rTOF contours extend into the right atrium and into subcutaneous fat past the apex. The LV contour is drawn in the left atrium on the most basal slice and at least one slice past the apex. The volumetric calculations are done using all contours, interpolating for missing slices

The most common error made by the algorithms, and likely the source of the largest volumetric discrepancies compared to gold standard contours, was when they generated ventricular contours in slices that were beyond the base or apex of the ventricles. This is likely because the UKBB data, on which the algorithms were primarily trained, are generally limited to and rarely extend beyond the ventricles. Given the RV dilation often found in patients with rTOF, and the desire to evaluate atrial performance, our practice is to extend the short-axis slices past the LV apex and into the atria, to avoid missing parts of the dilated RV apex and RV “shoulder” that extend basally past the tricuspid annulus (Fig. 6). Potential solutions include forcing ventricular contours to be in contiguous slices; use 3D datasets that may have clearer delineations of ventricular shape; or train with more datasets with slices that extend into the atria. We are actively working to solve this problem. Whereas this study was a head-to-head comparison, in clinical practice, this type of error would not occur as rapid manual correction would significantly improve the resultant volumetric measures. The results of this are shown in Supplemental Tables 7 and 8, where we manually removed the contours in excess slices, yielding improved %errors but overall similar results when comparing performance of MSN to MSN + rTOF. Some discrepancy between the results shown by spatial metrics and volumetric results can be related to the fact that the spatial metrics are less affected by having a single slice contoured as ventricular despite their being past the apex or base; the volume calculations are increased significantly by this because the volume is calculated by interpolating between slices (Fig. 6). Because the ventricular volume interpolates between distant slices, this causes significant increase in calculated ventricular volumes despite only modest changes to spatial metrics. This also reinforces the idea that when training a CNN, care should be given to maintaining data diversity because CNNs function best on the same kinds of data on which they were trained [37, 38].

### Limitations

The data used were clinical data and thus were not subjected to extra data cleaning and revisions. We specifically chose clinical data because our goal was to use real-world datasets instead of curated ones.

In the current form, this algorithm was limited to CMR studies where ES and ED were in the same cardiac phase for both the RV and LV. Given that this is not always the case (especially in rTOF where there can be right bundle branch block and other causes of phase misalignment), this represents a limitation of the current algorithm for use in clinical practice.

We performed this study on rTOF cases as this is the most common indication for congenital CMR. However, in the spectrum of congenital heart disease, the cardiac structure in rTOF is likely more similar to structurally normal hearts than other types of pathology, e.g., single ventricle disease or L-transposition of the great arteries, other common indications for congenital CMR. Thus, it is possible that a similar retraining strategy may not be as successful in those cases of more complex disease.

### Conclusions

We showed that a CNN, developed for structurally normal hearts, was able to be adapted to use in rTOF with a relatively small number of training cases, with acceptable but not ideal spatial and volumetric performance compared to manually drawn contours. rTOF is the most common indication for congenital CMR, so these findings support the development of contouring tools to increase efficiency of the clinical and research workflows for rTOF CMR. This work should be extended to other forms of congenital heart disease where the structural abnormalities are more extreme compared to structurally normal hearts. Aspects of this work were also rapidly translated into clinical use.

## Supplementary Information

Below is the link to the electronic supplementary material.

Electronic supplementary material 1 (DOCX 58 kb)

## Data Availability

Anonymized rTOF datasets will be made available with an appropriate data use agreement. The standard CNN-based algorithm is available in Circle cvi42 version 5.9. The ML algorithm used in MSN + rTOF is not available at the moment as the version in Circle cvi42 5.11 is more advanced. The code used is proprietary.

## Abbreviations

AVD: Average Hausdorff distance
BSA: Body surface area
bSSFP: Balanced steady-state free precession
CMR: Cardiovascular magnetic resonance
CNN: Convolutional neural network
DSC: Dice Similarity Coefficient
Endo: Endocardium
Epi: Epicardium
HD: Hausdorff distance
IQR: Interquartile range
LV: Left ventricle
MICCAI: Medical Image Computing and Computer Assisted Intervention
MSN: Mostly structurally normal
MSN + rTOF: Mostly structurally normal plus repaired tetralogy of Fallot
rTOF: Repaired tetralogy of Fallot
RV: Right ventricle
UKBB: United Kingdom Biobank
