# Supplementary material for: Retraining Convolutional Neural Networks for Specialized Cardiovascular Imaging Tasks: Lessons from Tetralogy of Fallot
Source: Pediatr Cardiol. 2021 Jan 4;42(3):578–89. doi: 10.1007/s00246-020-02518-5 (PMC7990832; doi:10.1007/s00246-020-02518-5)
Supplement: Supplementary file 1 — Electronic supplementary material 1 (DOCX 58 kb) [file 246_2020_2518_MOESM1_ESM.docx]

# Supplemental Table 1. Spatial performance of the Mostly Structurally Normal (MSN) algorithm on repaired tetralogy of Fallot (rTOF) training dataset.

| Evaluation Metric | Cardiac phase | N | LV Endo | LV Epi | RV Endo | LV Endo vs. LV Epi | LV Endo vs. RV Endo | LV Epi vs. RV Endo |
| --- | --- | --- | --- | --- | --- | --- | --- | --- |
| Dice Similarity Coefficient (DSC) (unitless) | Diastole | 57 | 0.884 (0.836-0.918) | 0.880 (0.848-0.913) | 0.830 (0.800-0.858) | 0.183 | **<0.0001** | **0.0001** |
|  | Systole | 29 | 0.800 (0.766-0.866) | 0.843 (0.807-0.879) | 0.745 (0.671-0.814) | 0.076 | **0.0076** | **<0.0001** |
| Hausdorff Distance (HD) (pixels) | Diastole | 57 | 3.84 (3.00-6.00) | 5.29 (3.62-7.69) | 12.7 (9.55-16.2) | 0.148 | **<0.0001** | **<0.0001** |
|  | Systole | 29 | 3.90 (3.09-5.54) | 6.35 (3.81-7.59) | 13.9 (10.8-18.4) | 0.147 | **<0.0001** | **<0.0001** |
| Average Hausdorff Distance (AVD) (pixels) | Diastole | 57 | 1.42 (1.16-2.78) | 2.23 (1.67-3.60) | 3.97 (2.52-5.68) | **0.011** | **<0.0001** | **<0.0001** |
|  | Systole | 29 | 1.61 (1.32-2.53) | 2.96 (1.66-3.50) | 4.86 (2.98-6.89) | **0.026** | **<0.0001** | **0.0076** |

This table shows the performance of the Mostly Structurally Normal (MSN) CNN algorithm on the repaired tetralogy of Fallot (rTOF) training dataset using spatial metrics. Data shown are median and interquartile ranges. Wilcoxon signed-rank tests and Friedman tests with Dunn’s multiple comparison tests were used as appropriate. Performance of the MSN CNN was superior for the LV endo and LV epi compared to RV endo for all metrics.

# Supplemental Table 2. Volumetric performance of the Mostly Structurally Normal (MSN) algorithm on repaired tetralogy of Fallot (rTOF) training dataset.

| Evaluation Metric | N | LV % error | RV % error | p-value |
| --- | --- | --- | --- | --- |
| EDV | 57 | 7.35 (3.285-15.21) | 10.70 (5.810-21.50) | 0.1278 |
| ESV | 57 | 13.62 (4.415-23.39) | 16.08 (9.990-33.87) | **0.0376** |
| EF | 57 | 10.34 (4.035-18.70) | 15.94 (4.400-36.07) | **0.0209** |

This table shows the performance of the Mostly Structurally Normal (MSN) CNN algorithm on the repaired tetralogy of Fallot (rTOF) training dataset using volumetrics. Data shown are median and interquartile ranges. Wilcoxon matched-pairs signed-rank tests were used. As expected, the MSN CNN was worse at ESV and EF calculations for the RV as compared to the LV.

# Supplemental Table 3. Spatial performance of the MSN+rTOF algorithm on the rTOF testing dataset, compared to the MSN algorithm.

| Evaluation Metric | Cardiac phase | Contour | N | MSN | MSN+rTOF | p-value (Wilcoxon rank-sum) | How many cases improved from MSN to MSN+rTOF |
| --- | --- | --- | --- | --- | --- | --- | --- |
| DSC (unitless) | End Diastole | LV Endo | 30 | 0.881 (0.818, 0.920) | 0.903 (0.875, 0.920) | **0.0248** | 18 |
|  |  | LV Epi | 30 | 0.883 (0.809, 0.909) | 0.905 (0.881, 0.937) | **<0.0001** | 27 |
|  |  | RV Endo | 30 | 0.850 (0.783, 0.886) | 0.894 (0.855, 0.907) | **<0.0001** | 27 |
|  | End Systole | LV Endo | 30 | 0.781 (0.721, 0.882) | 0.840 (0.776, 0.885) | 0.171 | 17 |
|  |  | LV Epi | 30 | 0.860 (0.793, 0.896) | 0.893 (0.860, 0.921) | **0.0003** | 23 |
|  |  | RV Endo | 30 | 0.775 (0.650, 0.816) | 0.825 (0.785, 0.864) | **<0.0001** | 26 |
| Hausdorff Distance (pixels) | End Diastole | LV Endo | 30 | 4.64 (2.84, 6.85) | 3.42 (2.45, 5.26) | 0.4045 | 15 |
|  |  | LV Epi | 30 | 6.30 (3.45, 9.26) | 3.82 (2.41, 5.73) | **<0.0001** | 26 |
|  |  | RV Endo | 30 | 12.5 (9.35, 16.0) | 9.28 (7.73, 11.7) | **<0.0001** | 27 |
|  | End Systole | LV Endo | 30 | 3.62 (3.05, 4.50) | 3.44 (2.52, 4.18) | 0.2621 | 17 |
|  |  | LV Epi | 30 | 4.85 (3.57, 7.44) | 4.06 (3.08, 5.76) | **0.0345** | 20 |
|  |  | RV Endo | 30 | 13.5 (10.2, 18.4) | 9.80 (7.65, 13.5) | **0.0004** | 24 |
| AVD (pixels) | End Diastole | LV Endo | 30 | 1.48 (1.14, 2.76) | 1.47 (0.957, 2.32) | 0.8872 | 14 |
|  |  | LV Epi | 30 | 2.53 (1.47, 4.47) | 1.49 (0.949, 2.53) | **<0.0001** | 26 |
|  |  | RV Endo | 30 | 3.34 (2.47, 6.06) | 2.29 (1.80, 3.23) | **<0.0001** | 27 |
|  | End Systole | LV Endo | 30 | 1.54 (1.25, 1.85) | 1.41 (1.07, 1.85) | 0.6120 | 16 |
|  |  | LV Epi | 30 | 2.22 (1.46, 3.29) | 1.52 (1.13, 2.22) | **0.0030** | 24 |
|  |  | RV Endo | 30 | 4.33 (2.79, 7.05) | 3.09 (2.13, 3.98) | **0.0087** | 22 |

This table shows the performance of the MSN and MSN+rTOF algorithms on the repaired tetralogy of Fallot (rTOF) testing dataset using spatial metrics. Data shown are median and interquartile ranges. Wilcoxon signed-rank tests were used. LV epi and RV endo contours improved from MSN to MSN+rTOF in all three spatial evaluation metrics (DSC, HD, AVD), with LV endo also having an improved DSC. The number of individual cases that improved from MSN to MSN+rTOF are also shown.

# Supplemental Table 4. Volumetric performance of the MSN+rTOF algorithm on the rTOF testing dataset, compared to the MSN algorithm.

|  | Correlation to manual | | | Bland-Altman analyses | | %Error | | |  |
| --- | --- | --- | --- | --- | --- | --- | --- | --- | --- |
| Volumetric Measure | MSN | MSN+rTOF | p-value of difference in correlation coefficient | MSN Bias SD and 95% Limits (mL or %) | MSN+rTOF Bias SD and 95% Limits (mL or %) | MSN %Error (Median, IQR) | MSN+rTOF %Error (Median, IQR) | p-value | Cases that improved from MSN to MSN+rTOF |
| LV EDV | 1.003 | 1.090 | 0.2656 | 5.07 (-47.87, 58.01) | 12.68 (-14.92, 40.27) | 6.14 (2.66, 19.25) | 11.10 (3.95, 21.91) | 0.146 | 9 |
| LV ESV | 0.8623 | 0.9293 | 0.6269 | 2.50 (-81.24, 86.25) | 17.38 (-41.54, 76.29) | 9.04 (5.04, 42.98) | 17.30 (7.68, 36.14) | 0.6263 | 13 |
| LV EF | 0.0893 | 0.4982 | 0.4318 | -2.08 (-50.35, 46.18) | -8.12 (-61.99, 45.75) | 6.64 (2.79, 22.89) | 7.46 (4.63, 24.60) | 0.7151 | 12 |
| LV ED mass | 0.7379 | 0.7036 | 0.7503 | 19.40 (-40.05, 78.85) | 4.58 (-40.97, 50.13) | 31.83 (5.60, 58.45) | 14.43 (8.93, 31.48) | **0.0248** | 20 |
| RV EDV | 0.8078 | 0.931 | **0.0459** | -10.98 (-49.52, 27.56) | 3.09 (-16.57, 22.74) | 11.39 (5.01, 20.94) | 8.10 (3.60, 11.81) | **0.0022** | 23 |
| RV ESV | 0.7213 | 0.8526 | 0.2711 | -6.00 (-80.97, 68.98) | 14.82 (-28.67, 58.32) | 11.53 (5.49, 29.44) | 14.89 (7.60, 32.66) | 0.5028 | 16 |
| RV EF | 0.6012 | 0.04478 | 0.3465 | -14.10 (-89.25, 61.04) | -17.37 (-76.68, 41.93) | 14.36 (4.10, 18.05) | 11.04 (5.66, 20.41) | 0.3818 | 17 |

This table shows the performance of the MSN and MSN+rTOF algorithms on the repaired tetralogy of Fallot (rTOF) testing dataset using volumetrics. Data shown are median and interquartile ranges. Wilcoxon signed-rank tests were used. For RV EDV, MSN+rTOF had improved correlation and %error compared to MSN, while MSN+rTOF LV ED mass was also improved. The number of individual cases that improved from MSN to MSN+rTOF are also shown.

# Supplemental Table 5. Spatial performance of the MSN+rTOF algorithm on the rTOF testing dataset, compared to intra- and interrater contours.

| Evaluation Metric | Cardiac phase | Contour | N | MSN+rTOF | Intrarater | Interrater | p-value (Wilcoxon rank-sum)  MSN+rTOF vs. Intrarater | p-value (Wilcoxon rank-sum)  MSN+rTOF vs. Interrater |
| --- | --- | --- | --- | --- | --- | --- | --- | --- |
| DSC (unitless) | End Diastole | LV Endo | 15 | 0.897 (0.870-0.925) | 0.905 (0.883-0.937) | 0.913 (0.877-0.930) | 0.639 | 0.330 |
|  |  | LV Epi | 15 | 0.898 (0.873-0.935) | 0.918 (0.877-0.935) | 0.911 (0.890-0.925) | 1.00 | 0.489 |
|  |  | RV Endo | 15 | 0.888 (0.835-0.903) | 0.899 (0.877-0.910) | 0.899 (0.866-0.928) | 0.303 | 0.188 |
|  | End Systole | LV Endo | 15 | 0.823 (0.736-0.885) | 0.850 (0.769-0.882) | 0.852 (0.791-.894) | 0.978 | 0.151 |
|  |  | LV Epi | 15 | 0.895 (0.863-0.915) | 0.899 (0.860-0.916) | 0.906 (0.868-0.925) | 0.561 | 0.762 |
|  |  | RV Endo | 15 | 0.814 (0.765-0.848) | 0.806 (0.765-0.869) | 0.818 (0.773-0.876) | 0.330 | 0.107 |
| Hausdorff Distance (pixels) | End Diastole | LV Endo | 15 | 3.79 (2.46-4.92) | 3.51 (2.20-4.62) | 3.51 (2.30-5.25) | 0.151 | 0.167 |
|  |  | LV Epi | 15 | 3.48 (2.44-6.00) | 4.09 (2.91-6.17) | 3.57 (2.44-5.59) | 0.847 | 0.135 |
|  |  | RV Endo | 15 | 9.87 (8.10-13.4) | 8.00 (6.46-12.1) | 9.74 (7.85-11.6) | 0.252 | 0.359 |
|  | End Systole | LV Endo | 15 | 3.53 (2.53-4.06) | 3.07 (2.38-3.94) | 2.85 (2.21-3.76) | 0.229 | **0.010** |
|  |  | LV Epi | 15 | 4.15 (3.34-7.24) | 3.05 (2.47-6.57) | 2.56 (2.30-4.78) | 0.359 | **0.0004** |
|  |  | RV Endo | 15 | 11.1 (7.15-14.7) | 11.2 (7.30-15.8) | 9.43 (7.83-14.6) | 0.454 | 0.679 |
| AVD (pixels) | End Diastole | LV Endo | 15 | 1.49 (1.17-2.17) | 1.55 (1.00-2.04) | 1.20 (0.962-1.94) | 0.720 | 0.055 |
|  |  | LV Epi | 15 | 1.34 (0.919-2.66) | 1.56 (1.28-2.78) | 1.09 (0.917-1.97) | 0.359 | 0.083 |
|  |  | RV Endo | 15 | 2.31 (2.05-3.53) | 2.10 (1.68-3.24) | 2.51 (1.93-4.02) | 0.169 | 0.679 |
|  | End Systole | LV Endo | 15 | 1.57 (1.15-1.86) | 1.34 (1.25-1.76) | 1.17 (0.886-1.50) | 0.679 | **0.041** |
|  |  | LV Epi | 15 | 1.46 (1.13-2.76) | 1.18 (1.09-2.89) | 1.03 (0.869-1.56) | 0.978 | **0.0001** |
|  |  | RV Endo | 15 | 3.62 (2.20-4.22) | 3.79 (2.33-5.75) | 2.82 (2.22-4.42) | 0.208 | 0.188 |

This table shows the spatial performance MSN+rTOF retrained CNN algorithm compared to intra- and interrater contouring for the 15 cases reviewed. Data shown are median and interquartile ranges. Wilcoxon signed-rank tests were used.

# Supplemental Table 6. Volumetric performance of the MSN+rTOF algorithm on the rTOF testing dataset, compared to intra- and interrater contours.

|  | MSN+rTOF %error | Intrarater %error | Interrater %error | p-value (Wilcoxon rank-sum)  MSN+rTOF vs. Intrarater | p-value (Wilcoxon rank-sum)  MSN+rTOF vs. Interrater |
| --- | --- | --- | --- | --- | --- |
| LV EDV | 10.89 (3.24, 25.18) | 5.41 (2.27, 7.09) | 6.63 (2.95, 9.67) | **0.0054** | **0.0413** |
| LV ESV | 19.45 (8.80, 52.25) | 5.65 (2.98, 12.87) | 22.23 (8.14, 31.91) | **0.0006** | 0.5614 |
| LV EF | 13.18 (4.88, 28.13) | 3.56 (0.72, 6.39) | 12.08 (4.06, 12.75) | **0.0084** | 0.6387 |
| LV ED mass | 12.85 (8.82, 34.94) | 13.68 (8.87, 14.86) | 11.23 (5.28, 18.44) | 0.4456 | 0.1876 |
| RV EDV | 8.70 (4.11, 14.58) | 6.04 (5.56, 9.10) | 6.54 (1.86, 10.22) | 0.1205 | 0.0554 |
| RV ESV | 15.84 (7.46, 26.33) | 11.93 (4.04, 17.90) | 12.48 (5.74, 26.64) | 0.3817 | 0.4887 |
| RV EF | 10.47 (3.95, 17.28) | 10.69 (6.32, 13.66) | 8.92 (3.26, 14.20) | 0.7615 | 0.1646 |

This table shows the volumetric performance MSN+rTOF retrained CNN algorithm compared to intra- and interrater contouring for the 15 cases reviewed. Data shown are median and interquartile ranges.

# Supplemental Table 7. Spatial performance of the MSN+rTOF algorithm on the rTOF testing dataset, compared to the MSN algorithm, when algorithm-generated contours are removed from slices where there were no manual contours.

| Evaluation Metric | Cardiac phase | Contour | N | MSN | MSN+rTOF | p-value (Wilcoxon rank-sum) | How many cases improved from MSN to MSN+rTOF |
| --- | --- | --- | --- | --- | --- | --- | --- |
| DSC (unitless) | End Diastole | LV Endo | 30 | 0.902 (0.844, 0.922) | 0.916 (0.889, 0.928) | **0.0066** | 21 |
|  |  | LV Epi | 30 | 0.892 (0.845, 0.923) | 0.915 (0.893, 0.943) | **<0.0001** | 29 |
|  |  | RV Endo | 30 | 0.862 (0.792, 0.900) | 0.909 (0.872, 0.923) | **<0.0001** | 29 |
|  | End Systole | LV Endo | 30 | 0.836 (0.769, 0.882) | 0.871 (0.815, 0.885) | 0.2129 | 17 |
|  |  | LV Epi | 30 | 0.881 (0.856, 0.912) | 0.914 (0.881, 0.936) | **<0.0001** | 27 |
|  |  | RV Endo | 30 | 0.799 (0.737, 0.841) | 0.860 (0.833, 0.893) | **<0.0001** | 27 |
| Hausdorff Distance (pixels) | End Diastole | LV Endo | 30 | 4.640 (2.841, 6.850) | 3.418 (2.449, 5.262) | 0.4045 | 15 |
|  |  | LV Epi | 30 | 5.439 (3.455, 9.068) | 3.819 (2.409, 5.731) | **0.0002** | 25 |
|  |  | RV Endo | 30 | 12.224 (8.961, 16.046) | 9.191 (7.176, 11.664) | **<0.0001** | 27 |
|  | End Systole | LV Endo | 30 | 3.622 (3.053, 4.502) | 3.439 (2.521, 4.184) | 0.2621 | 17 |
|  |  | LV Epi | 30 | 4.619 (3.568, 7.442) | 3.655 (2.877, 5.304) | **0.0004** | 24 |
|  |  | RV Endo | 30 | 12.767 (10.167, 17.221) | 9.587 (7.554, 13.075) | **<0.0001** | 24 |
| AVD (pixels) | End Diastole | LV Endo | 30 | 1.477 (1.137, 2.759) | 1.470 (0.957, 2.324) | 0.8872 | 14 |
|  |  | LV Epi | 30 | 2.254 (1.467, 4.153) | 1.488 (0.949, 2.529) | **<0.0001** | 25 |
|  |  | RV Endo | 30 | 3.335 (2.311, 5.662) | 2.257 (1.605, 2.916) | **<0.0001** | 27 |
|  | End Systole | LV Endo | 30 | 1.540 (1.250, 1.854) | 1.410 (1.074, 1.847) | 0.612 | 16 |
|  |  | LV Epi | 30 | 2.145 (1.460, 3.292) | 1.360 (1.117, 1.814) | **<0.0001** | 28 |
|  |  | RV Endo | 30 | 4.328 (2.786, 6.066) | 2.837 (2.064, 3.754) | **0.0003** | 24 |

This table shows the performance of the MSN and MSN+rTOF algorithms on the repaired tetralogy of Fallot (rTOF) testing dataset using spatial metrics. Data shown are median and interquartile ranges. Wilcoxon signed-rank tests were used. LV epi and RV endo contours improved from MSN to MSN+rTOF in all three spatial evaluation metrics (DSC, HD, AVD), with LV endo also having an improved DSC at end diastole. The number of individual cases that improved from MSN to MSN+rTOF are also shown.

# Supplemental Table 8. Volumetric performance of the MSN+rTOF algorithm on the rTOF testing dataset, compared to the MSN algorithm, when algorithm-generated contours are removed from slices where there were no manual contours.

|  | Correlation to manual | | | Bland-Altman analyses | | %Error | | |  |
| --- | --- | --- | --- | --- | --- | --- | --- | --- | --- |
| Volumetric Measure | MSN | MSN+rTOF | p-value of difference in correlation coefficient | MSN Bias SD and 95% Limits (mL or %) | MSN+rTOF Bias SD and 95% Limits (mL or %) | MSN %Error (Median, IQR) | MSN+rTOF %Error (Median, IQR) | p-value | Cases that improved from MSN to MSN+rTOF |
| LV EDV | 1.065 | 1.109 | 0.2714 | 0.91 (-16.81, 18.62) | 6.87 (-7.47, 21.20) | 5.43 (2.67, 10.66) | 7.79 (3.10, 12.99) | 0.2621 | 11 |
| LV ESV | 0.9733 | 1.023 | 0.4965 | -10.28 (-45.10, 24.53) | 6.65 (-25.06, 38.36) | 6.48 (3.12, 13.91) | 12.07 (6.34, 17.95) | 0.3085 | 14 |
| LV EF | 0.9036 | 0.7413 | 0.5102 | 6.27 (-10.34, 22.88) | -0.59 (-24.91, 23.72) | 4.73 (2.65, 12.91) | 3.45 (1.68, 9.56) | 0.2206 | 18 |
| LV ED mass | 0.7935 | 0.7073 | 0.3515 | 12.13 (-28.18, 52.44) | -2.18 (-41.17, 36.81) | 12.76 (5.60, 33.45) | 12.91 (8.74, 19.73) | 0.1893 | 17 |
| RV EDV | 0.8176 | 0.932 | **0.0392** | -11.78 (-37.82, 14.27) | 0.19 (-17.68, 18.06) | 11.34 (5.01, 19.55) | 5.95 (1.52, 10.14) | **0.0002** | 24 |
| RV ESV | 0.8308 | 0.9249 | 0.2975 | -14.76 (-71.56, 42.03) | 4.48 (-23.06, 32.02) | 12.23 (5.49, 25.81) | 11.78 (6.45, 15.45) | 0.0879 | 19 |
| RV EF | 1.053 | 0.7318 | 0.4047 | 0.00 (0.00, 0.00) | 0.00 (0.00, 0.00) | 12.68 (4.76, 19.04) | 8.81 (3.49, 11.94) | 0.1153 | 18 |

This table shows the performance of the MSN and MSN+rTOF algorithms on the repaired tetralogy of Fallot (rTOF) testing dataset using volumetrics, corrected for when the algorithms created contours in slices where there were no manual contours. Data shown are median and interquartile ranges. Wilcoxon signed-rank tests were used. For RV EDV, MSN+rTOF had improved correlation and %error compared to MSN. The number of individual cases that improved from MSN to MSN+rTOF are also shown.
